# Supplementary material for: ProtGPT2 is a deep unsupervised language model for protein design
Source: Nat Commun. 2022 Jul 27;13:4348. doi: 10.1038/s41467-022-32007-7 (PMC9329459; doi:10.1038/s41467-022-32007-7)
Supplement: Supplementary file 1 — Supplementary Information [file 41467_2022_32007_MOESM1_ESM.pdf]

Supporting Information for:

ProtGPT2 is a deep unsupervised language model for protein design

Noelia Ferruz[1,2*], Steffen Schmidt[3], Birte Höcker[1]

[1]Department of Biochemistry, University of Bayreuth, Bayreuth, Germany.

[2]Current address: Institute of Informatics and Applications, University of Girona, Girona, Spain

[3]Computational Biochemistry. University of Bayreuth, 95447 Bayreuth, Germany.

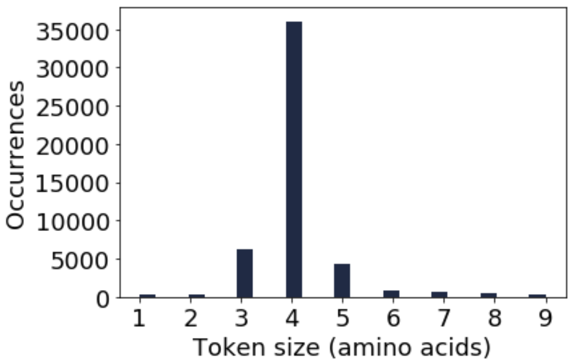

Supplementary Figure 1: Histogram of token sizes in the vocabulary. Number of tokens in the vocabulary that show a certain amino acid length. Most tokens consist of tetramers.

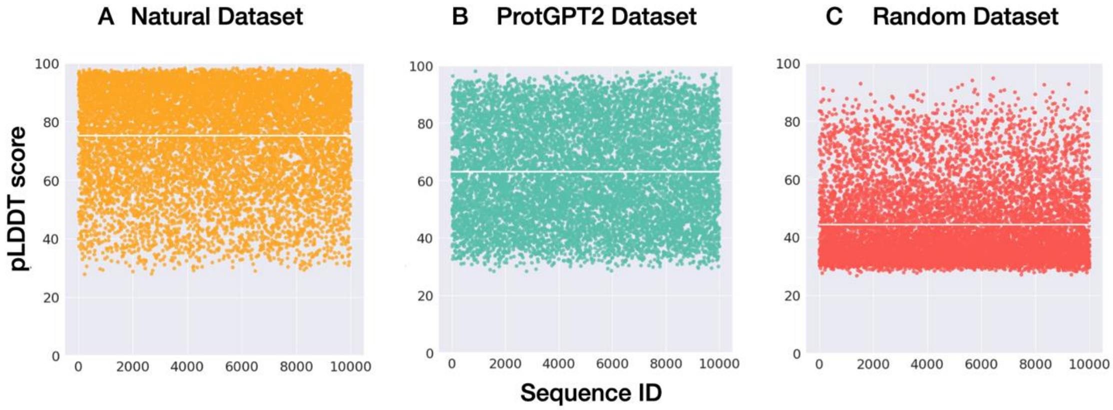

Supplementary Figure 2: AlphaFold score values across datasets. pLDDT values for each sequence in the ProtGPT2 **(a)**, natural **(b)** and random **(c)** datasets. The values were computed averaging over each residue in the predicted structure. White lines depict the average values for each dataset. The coloring method applies to all figures in this manuscript (yellow; natural dataset; cyan: ProtGPT2 dataset; salmon: random dataset)

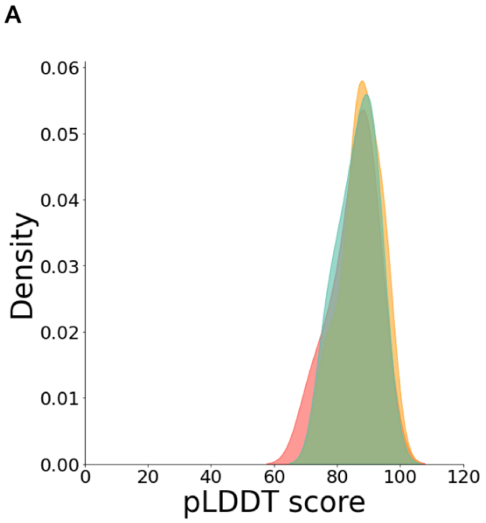

Supplementary Figure 3: Simulation results for three datasets. Distribution of pLDDT scores for each of the 12 simulations in the three datasets. Simulated sequences have similar pLDDT values.

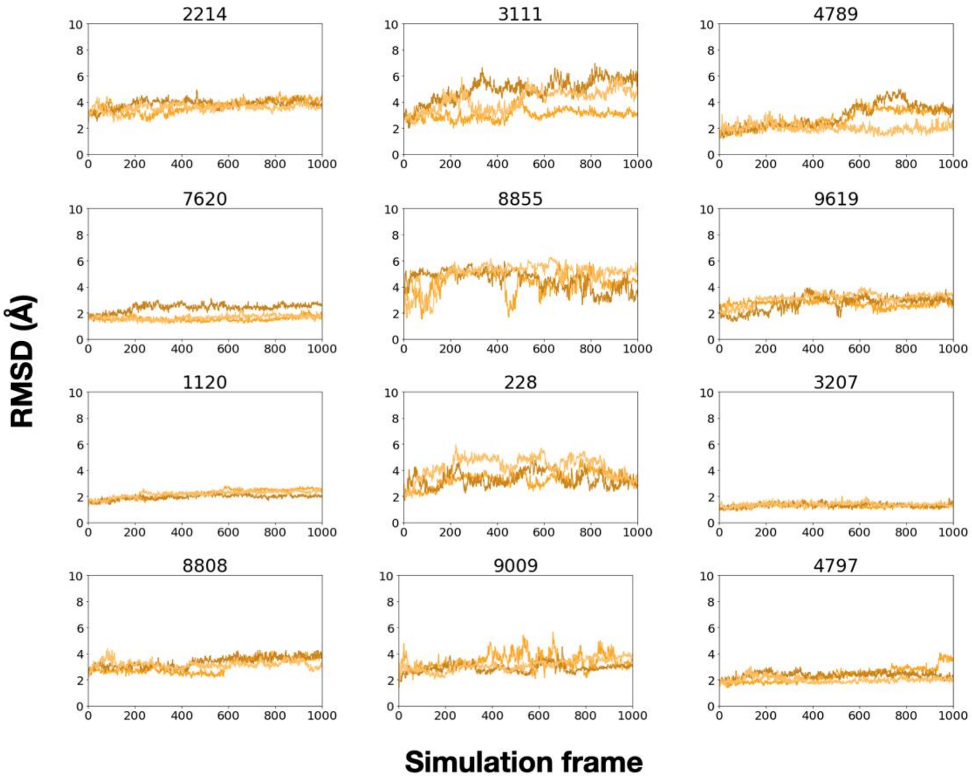

Supplementary Figure 4: Explicit RMSD values for each of the simulated trajectories in the natural dataset. The three independent trajectories are shown in a different shade of yellow. The simulations ran for 1000 frames, which are equivalent to 100 ns.

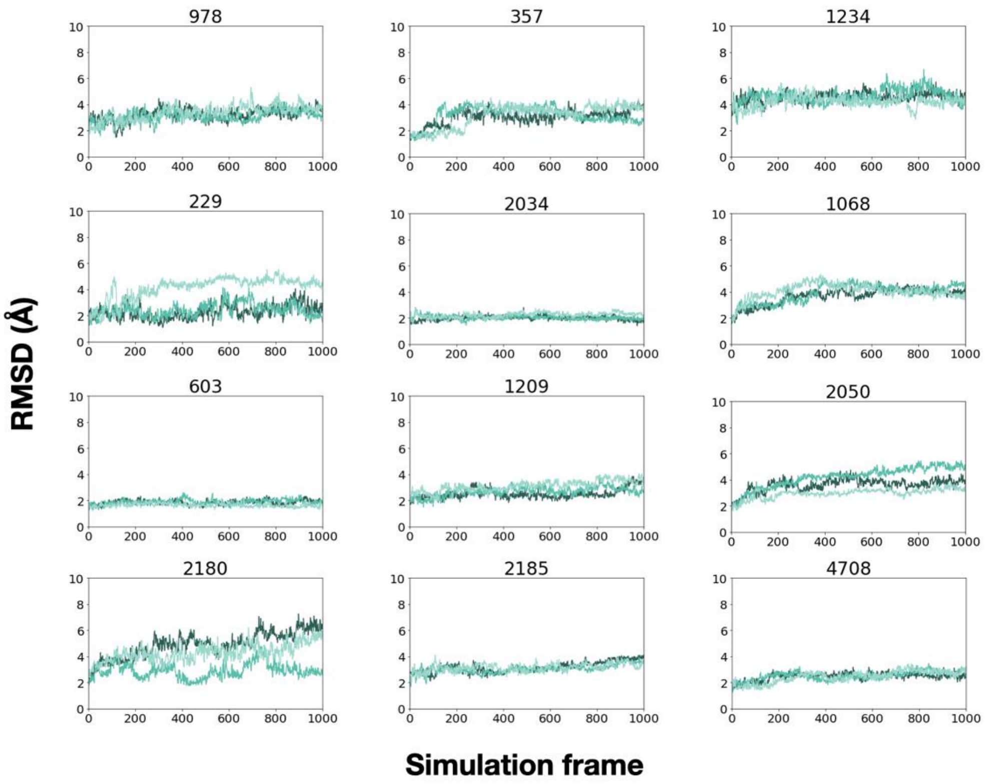

Supplementary Figure 5: Explicit RMSD values for each of the simulated trajectories in the ProtGPT2 dataset. The three independent trajectories are shown in a different shade of blue. The simulations ran for 1000 frames, which are equivalent to 100 ns.

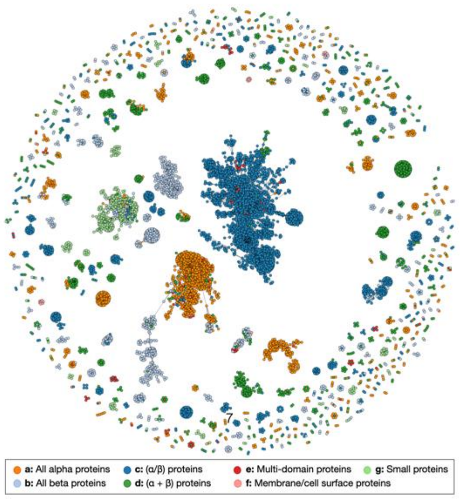

Supplementary Figure 6: The protein sequence space represented with SCOP95 sequences. Each node in the network represents a protein sequence, which is linked to others when they have an alignment of at least 20 amino acids in length and with a HHsearch probability over 70%. Each of the seven major SCOP classes is depicted in a different color. The graph contains 13,201 vertices and 97,705 edges.
