# Supplementary material for: ProtGPT2 is a deep unsupervised language model for protein design
Source: Nat Commun. 2022 Jul 27;13:4348. doi: 10.1038/s41467-022-32007-7 (PMC9329459; doi:10.1038/s41467-022-32007-7)
Supplement: Supplementary file 2 — Reporting Summary [file 41467_2022_32007_MOESM2_ESM.pdf]

nature portfolio

Corresponding author(s): Noelia Ferruz

Last updated by author(s): Jul 8, 2022

Reporting Summary

Nature Portfolio wishes to improve the reproducibility of the work that we publish. This form provides structure for consistency and transparency in reporting. For further information on Nature Portfolio policies, see our Editorial Policies and the Editorial Policy Checklist.

Statistics

For all statistical analyses, confirm that the following items are present in the figure legend, table legend, main text, or Methods section.

n/a	Confirmed	
☐	☒	The exact sample size (*n*) for each experimental group/condition, given as a discrete number and unit of measurement
☒	☐	A statement on whether measurements were taken from distinct samples or whether the same sample was measured repeatedly
☐	☒	The statistical test(s) used AND whether they are one- or two-sided<br>*Only common tests should be described solely by name; describe more complex techniques in the Methods section.*
☒	☐	A description of all covariates tested
☒	☐	A description of any assumptions or corrections, such as tests of normality and adjustment for multiple comparisons
☐	☒	A full description of the statistical parameters including central tendency (e.g. means) or other basic estimates (e.g. regression coefficient) AND variation (e.g. standard deviation) or associated estimates of uncertainty (e.g. confidence intervals)
☐	☒	For null hypothesis testing, the test statistic (e.g. *F*, *t*, *r*) with confidence intervals, effect sizes, degrees of freedom and *P* value noted<br>*Give P values as exact values whenever suitable.*
☒	☐	For Bayesian analysis, information on the choice of priors and Markov chain Monte Carlo settings
☒	☐	For hierarchical and complex designs, identification of the appropriate level for tests and full reporting of outcomes
☒	☐	Estimates of effect sizes (e.g. Cohen's *d*, Pearson's *r*), indicating how they were calculated

Our web collection on statistics for biologists contains articles on many of the points above.

Software and code

Policy information about availability of computer code

Data collection	The data was processed with the dataset package from HuggingFace version 1.16.1.
Data analysis	The data was trained with the HuggingFace transformer library version 4.14.1. The sequences were analyzed with PSIPRED v4.0, IUPRED3, AlphaFold2 ColabFold batch v1.2, the Rosetta Software Suite v3.12. The simulations ran with ACEMD v3.

For manuscripts utilizing custom algorithms or software that are central to the research but not yet described in published literature, software must be made available to editors and reviewers. We strongly encourage code deposition in a community repository (e.g. GitHub). See the Nature Portfolio guidelines for submitting code & software for further information.

Data

Policy information about availability of data

All manuscripts must include a data availability statement. This statement should provide the following information, where applicable:

- Accession codes, unique identifiers, or web links for publicly available datasets
- A description of any restrictions on data availability
- For clinical datasets or third party data, please ensure that the statement adheres to our policy

The model weights are publicly available in the HuggingFace repository: https://huggingface.co/nferruz/ProtGPT2 and Zenodo: 10.5281/zenodo.6796843. The dataset for training is available at: https://huggingface.co/datasets/nferruz/UR50_2021_04. The three sequence datasets in this work are available at: https://huggingface.co/datasets/nferruz/dataset_fastas. The AlphaFold predictions for the three datasets are available at https://huggingface.co/datasets/nferruz/dataset_alphafold. The Uniref50 original database version 21_04 is available at https://ftp.uniprot.org/pub/databases/uniprot/previous_releases/release-2021_04/.

The Uniclust30 database version 2018_08 is available at http://gwdu111.gwdg.de/~compbiol/uniclust/2018_08/uniclust30_2018_08_hhsuite.tar.gz

Human research participants

Policy information about studies involving human research participants and Sex and Gender in Research.

Reporting on sex and gender	Not Applicable
Population characteristics	Not Applicable
Recruitment	Not Applicable
Ethics oversight	Not Applicable

Note that full information on the approval of the study protocol must also be provided in the manuscript.

Field-specific reporting

Please select the one below that is the best fit for your research. If you are not sure, read the appropriate sections before making your selection.

☒ Life sciences ☐ Behavioural & social sciences ☐ Ecological, evolutionary & environmental sciences

For a reference copy of the document with all sections, see nature.com/documents/nr-reporting-summary-flat.pdf

Life sciences study design

All studies must disclose on these points even when the disclosure is negative.

Sample size	A sample size of 10,000 sequences per dataset was chosen to allow for sufficient sequence diversity while allowing tractable posterior analyses (AlphaFold, IUPRED, Rosetta...)
Data exclusions	No data was excluded in this work.
Replication	We produced three independent replicas for each of the 36 sequences analyzed with molecular dynamics simulations. For the rest of the analyses one single prediction was performed for each of the independent sequences (AlphaFold, Rosetta, IUPRED)
Randomization	The selected dataset was created by randomly taking 100,000 sequences from Uniref50 with the Python library 'random' and further selecting 10,000 random sequences whose length distributions matched that of the ProtGPT2 dataset (<250 amino acids).<br>Random sequences were created by randomly concatenating the 25 amino acids with the Python library random to reach a randomly selected length matching the length distribution of the other two datasets.
Blinding	No blinding of different groups or replicas was needed to perform the analyses in this study.

Reporting for specific materials, systems and methods

We require information from authors about some types of materials, experimental systems and methods used in many studies. Here, indicate whether each material, system or method listed is relevant to your study. If you are not sure if a list item applies to your research, read the appropriate section before selecting a response.

Materials & experimental systems

n/a	Involved in the study
☒	☐ Antibodies
☒	☐ Eukaryotic cell lines
☒	☐ Palaeontology and archaeology
☒	☐ Animals and other organisms
☒	☐ Clinical data
☒	☐ Dual use research of concern

Methods

n/a	Involved in the study
☒	☐ ChIP-seq
☒	☐ Flow cytometry
☒	☐ MRI-based neuroimaging
